# Targeting the Gut–Brain Axis with Fecal Microbiota Transplantation: Considerations on a Potential Novel Treatment for Parkinson's Disease

**DOI:** 10.1002/mdc3.13621

**Published:** 2022-12-07

**Authors:** Maria Fiorella Contarino, Jacobus J. van Hilten, Ed J. Kuijper

**Affiliations:** ^1^ Department of Neurology Leiden University Medical Center Leiden The Netherlands; ^2^ Department of Neurology Haga Teaching Hospital The Hague The Netherlands; ^3^ Department of Medical Microbiology Leiden University Medical Center Leiden The Netherlands; ^4^ Center for Infectious Disease Control National Institute for Public Health and the Environment (Rijksinstituut voor Volksgezondheid en Milieu) Bilthoven The Netherlands

**Keywords:** Parkinson's disease, gastrointestinal system, microbiota, fecal microbiota transplantation, gut–brain axis

## Why the Gut?

The bidirectional communication between the gut and the central nervous system (CNS) has been identified as the “gut–brain axis.” Gastrointestinal symptoms occur in up to 80% of patients with Parkinson's disease (PD) and can precede the onset of motor symptoms by more than 20 years.^1^, ^2^ Lewy bodies and α‐synuclein pathology are found in 67% of enteric nervous system (ENS) and vagus nerve biopsies in PD compared with only 26% controls, even years before the onset of motor symptoms.^3^, ^4^

Evidence for increased intestinal inflammation and permeability is present also in newly diagnosed, untreated patients with PD and in mouse models of PD. Inflammation‐induced oxidative stress can be the substrate of α‐synuclein misfolding and aggregation in the gut.^5^, ^6^ The misfolded α‐synuclein could then reach the brain via the vagus nerve, as demonstrated in some animal models,^3^, ^7^, ^8^, ^9^, ^10^ and truncal vagotomy might reduce the risk of PD,^11^ although the evidence supporting this finding is still inconsistent.

All of this evidence suggests that the gut has a prominent role in the pathogenesis and progression of PD and that PD pathology may even be triggered in the gut. One of the most accredited hypotheses on PD pathology progression is the so‐called “dual–hit Braak hypothesis.^3^, ^12^ This postulates that α‐synuclein aggregation in sporadic PD is triggered by microbial products in the olfactory neurons and the enteric mucosal neurons, alternatively or simultaneously. Following on this, the “body‐first/brain‐first” hypothesis^13^ proposed 2 distinct phenotypes: one with pathology starting in the gut, successively involving the substantia nigra (“body‐first”), and one with pathology starting in the amygdala or olfactory bulb, following only later to the gut (“brain‐first”).

These and other similar theories based on the pathological distribution of Lewy bodies pathology have been widely criticized, starting from the role of Lewy bodies in the pathogenesis of PD. Other pathological, clinical, and neuroimaging observations point to a large variety in the spread of the pathological process across patients^14^, ^15^, ^16^; hypothetically, a different gut microbiota makeup could be one of the factors underlying this variety, opening avenues to new treatment strategies.

## The Gut Microbiota in PD

Healthy individuals host up to 400 of >2000 different bacterial species in the intestinal tract, in addition to archaea, viruses, and yeasts. The gut microbiota composition is established in the first 4 years of life and remains relatively stable in adult life, although it can be influenced by factors such as diet, stress, inflammation, and medications. The gut microbiota produces vitamins, short‐chain fatty acids (SCFAs), enzymes, and even neurotransmitters such as dopamine, acetylcholine, and serotonin. It influences immune regulation and might play a role in the development and progression of immune diseases.

A recent meta‐analysis of 10 microbiome data sets confirmed that PD gut microbiota is significantly different from controls and displays a relative abundance of proinflammatory, and a scarcity of anti‐inflammatory, bacterial species, with less SCFA producers.^17^ Specific bacterial taxa have been associated with the incidence and severity of PD motor symptoms or constipation.^18^, ^19^, ^20^ The proinflammatory activity of some Proteobacteria may represent the main cause of the intestinal barrier damage resulting in increased intestinal permeability. Studies on PD animal models confirm that the gut microbiota composition is determinant to induce α‐synuclein pathology, neuroinflammation, and the onset of the motor phenotype.^21^ Modifying gut microbiota in mice can decrease the severity of the pathological abnormalities and motor and gastrointestinal symptoms.^21^, ^22^ Recently, 2‐hydroxypyridine, a molecule correlated with the archeal species *Methanobrevibacter smithii*, was shown to promote α‐synuclein aggregation and exacerbate PD‐related motor symptoms and striatal degeneration in a transgenic mouse.^23^

All the aforementioned evidence strongly supports a correlation between a specific makeup of the gut microbiota and the pathophysiology of PD. Although some animal data may indicate that this correlation is causative, insufficient evidence is available in patients to exclude a mere consequential role.

Another recent finding is that gut bacterial tyrosine decarboxylases (TDCs) can decarboxylate levodopa (l‐dopa) to dopamine without being inhibited by carbidopa. By altering l‐dopa levels in the gut and blood, bacterial TDCs can thus possibly influence the development and severity of response fluctuations.^24^, ^25^

## Modification of Gut Microbiota as a Treatment Strategy for PD: Preliminary Considerations

### Is It Possible to Modify Gut Microbiota?

There are different strategies to modify gut microbiota composition, ranging from dietary interventions^26^ to treatment with dietary supplements, antibiotics, or genetically engineered microorganisms.^27^

Specific antibiotics may target proinflammatory bacteria, thus exerting an anti‐inflammatory, antiapoptotic, and antioxidant action that may result in neuroprotection.^28^ Treatments with prebiotics, probiotics, or symbiotics and the application of small molecules can foster the growth of bacteria producing SCFA with an anti‐inflammatory action. Unfortunately, the large variety of methods in probiotics studies and the lack of a correlation with the host original microbiota composition make it difficult to draw definitive conclusions, and a meta‐analysis, currently underway, could provide more information.^29^ This potential beneficial action is also not consistently supported by clinical observations, which could be attributed to an incomplete or only temporary alteration of the gut microbiota.

A more drastic strategy to modify gut microbiota is fecal microbiota transplantation (FMT). FMT implies the administration of a solution of fecal matter from a healthy donor to a host with the aim of replacing the recipient's gut microbial composition and metabolic activities.^30^, ^31^ The only approved indication of FMT is multiple recurrent and severe *Clostridioides difficile* infections (CDI), for which it is proven safe and effective.^31^

### Is It Meaningful to Modify Gut Microbiota?

Based on the available evidence and consequent hypotheses, it is tempting to speculate that modifying the gut microbiota could represent a strategy to affect the disease pathophysiology. A reduction of the proinflammatory agents and their products could result in less intestinal inflammation, with a consequent reduction of α‐synuclein aggregation in the ENS and therefore the CNS. This could be reflected in a reduction of severity of PD symptoms and a slowing of disease progression, especially when such intervention would be performed at an early disease stage.

If it would be demonstrated that, at least in some cases, the disease originates in the gut, modifying the microbiota in presymptomatic subjects may delay or even prevent the trigger to disease onset.

A low‐hanging fruit could be to improve l‐dopa metabolism and absorption in the gut acting on specific TDC‐producing bacterial species, which could result in a better effect and a reduction of l‐dopa–mediated motor complications.

Finally, an additional benefit could be the restoration of a normal defecation pattern.

### FMT for PD: Available Evidence of Efficacy

Several preliminary clinical observations on FMT for different neurological disorders,^32^ including PD,^33^, ^34^, ^35^, ^36^ are being published, and study protocols exploring this potential treatment for PD are being registered.^37^

So far, a total of 33 PD cases have been reported, all studied in open‐label fashion.^33^, ^34^, ^35^, ^36^ The reported patients presented with different clinical characteristics and were evaluated in different ways for variable follow‐up periods. Also, pretransplant strategies, FMT methodology, and administration route varied significantly among centers. In the majority of the cases, the results show subjective and objective improvement of motor symptoms and some nonmotor symptoms, including sleep, anxiety, and depression, to different extents and durations. In particular, improvement of constipation was an almost constant finding. These results need to be viewed critically considering that the evaluations were not standardized and not blinded, leaving room for a placebo effect.

### Safety Considerations

Mild and temporary adverse effects related to the FMT procedure are reported in 20% to 40% of the patients and include mild abdominal pain and diarrhea.^38^ However, in 0% to 5% of the cases, possibly related severe adverse events were reported, including aspiration pneumonia, septicemia, systemic inflammatory response syndrome, and upper gastrointestinal hemorrhage.^38^, ^39^, ^40^ Interestingly, the long‐term adverse effects of FMT are largely unknown, although the first data indicate long‐term safety,^41^ and large registers to evaluate long‐term outcomes are ongoing (NCT03325855).

Little is known about the incidence of adverse effects in patients undergoing FMT for indications other than CDI. Patients with PD would probably present a similar incidence of adverse effects, when excluding patients with severe dysphagia who could incur in a higher risk of aspiration. Among the few published cases, 1 patient with PD reported episodes of vasovagal presyncope needing hospital admission.^35^ In all the other cases, only mild transient adverse effects were reported.^34^, ^36^

One aspect to be considered in the PD population is that theoretically the radical microbiota replacement could even result in a deterioration of PD symptoms and altered l‐dopa response, depending on the specific composition of the donor microbiota. Therefore, the storage of patients' own feces for the purpose of autologous rescue FMT should always be considered.

### Potential Pitfalls in FMT Studies for PD

FMT is a complex treatment, and a large variability of methods is reported in different centers. Some of these factors my turn out to be of crucial importance for the treatment results in patients with PD.

One important aspect is the donor choice. Surely donors need to be rigorously screened following standardized protocols in accredited centers to guarantee the safety of the procedure. However, at this stage it is not known what would be the ideal microbiota composition to be transplanted to be most effective in PD. Although many studies show clear differences between the microbiota and metabolome of patients with PD and that of controls, only a minimal number of observations are constantly replicated in different cohorts compared with the great complexity and variability of the gut microbiota. The growing number of studies on the effect of specific bacteria, and especially microbial products, on experimental PD models will ultimately help define a desirable microbiota composition for FMT, although more complex microbiota–host interactions might also play a role. In addition, the donors are often young volunteers, and no effort is made to identify potential risk factors for the future development of neurodegenerative disorders. Ideally, donors should undergo a long‐term follow‐up themselves to monitor the onset of neurological disorders.

Different methods of collecting, transporting, processing, and storing donor feces are currently in use. Each of these steps might introduce alterations in the donor microbiota and metabolome that could potentially affect its efficacy and impair the comparability among different studies. Different administration routes (gastroscopy, colonoscopy, or even capsules) may be linked to a different risk profile but also affect the efficacy of the transplant when, for example, large intestinal flora are transplanted in the duodenum via gastroscopy.

Also, the pretreatment preparation of the receiver could impact the result. Currently, some centers prescribe antibiotics (usually vancomycin) and/or apply pretreatment bowel lavage to increase the FMT engraftment. These factors per se could be important in inducing changes in PD symptoms, albeit probably only in the short term.

Another unknown parameter is the duration of the induced changes; it could be hypothesized that, different from CDI treatment, both induction and maintenance treatments should be considered in the case of a progressive neurodegenerative disease.

Last but not least, similar to other treatment strategies, FMT could produce different effects in specific patient groups. Factors such as genetic predisposition, disease duration, and age might be critical. Similarly, it might be relevant to select patients based on the presence of constipation or maybe based on their pattern of response to l‐dopa, for example, selecting those needing very high dosages or conversely those reporting dyskinesia at a minimal dosage.

## Final Considerations

The gut–brain axis is gaining increasing attention not only in the scientific community but also among patient organizations, and it is not unusual that patients manifest a strong trust in a beneficial effect of microbiota‐modifying strategies. For this reason, many take the initiative to follow dietary restrictions or use food supplements and are ready to undergo more invasive treatments if offered.

There are currently no treatments proven effective in modifying the disease course, and the available literature gives ground to the enthusiasm for these new strategies and justifies designing pilot studies and clinical trials to further explore all potential benefits. However, the danger of blindly following the hype is to give our patients false hopes and to push them toward invasive treatments that might be inefficacious or even detrimental.

At this very early stage, it is our responsibility as clinicians and researchers to rigorously test all the hypotheses in controlled experimental settings but also to remain critical about the preliminary encouraging reports and use caution when considering all the expected benefits and risks of these strategies.

## Author Roles

(1) Research Project: A. Conception, B. Organization, C. Execution; (2) Manuscript Preparation: A. Writing of the First Draft, B. Review and Critique.

M.F.C.: 1A, 1B, 1C, 2A

J.J.v.H.: 2B

E.J.K.: 2B

## Disclosures

**Ethical Compliance Statement:** The authors confirm that the approval of an institutional review board was not necessary for this work, nor was informed consent. We confirm that we have read the Journal's position on issues involved in ethical publication and affirm that this work is consistent with those guidelines.

**Funding Sources and Conflicts of Interest:** The authors have no conflicts of interest related to this work. No funding was received for this work.

**Financial Disclosures for Previous 12 Months:** J.J.v.H. reports grants from AbbVie and research support from the Centre of Human Drug Research. E.J.K. reports an unrestricted grant from Vedanta. M.F.C. reports the following in the past year: advisory board memberships with Medtronic (fees to institution) and AbbVie, independent consultancy with Medtronic for research and educational issues (fees to institution), and speaking fees from European Continuing Medical Training (ECMT) Continuing Medical Education (CME activity) and Boston Scientific (fees to institution).
